# Triglyceride-glucose index as a prognostic marker after ischemic stroke or transient ischemic attack: a prospective observational study

**DOI:** 10.1186/s12933-022-01695-2

**Published:** 2022-11-30

**Authors:** Takao Hoshino, Takafumi Mizuno, Kentaro Ishizuka, Shuntaro Takahashi, Satoko Arai, Sono Toi, Kazuo Kitagawa

**Affiliations:** Department of Neurology, Tokyo Women’s Medical University Hospital, 8-1, Kawada-Cho, Shinjuku-Ku, Tokyo, 162-8666 Japan

**Keywords:** Atherosclerosis, Cerebrovascular disease, Insulin resistance, Prognosis, Stroke, Triglyceride-glucose index

## Abstract

**Background:**

Triglyceride-glucose (TyG) index has been proposed as a simple and credible surrogate for insulin resistance and an independent predictor of cardiovascular outcomes. Due to lack of data on TyG index in stroke, we aimed to evaluate the predictive value of the index for recurrent vascular event risk among stroke patients.

**Methods:**

This was a prospective observational study, in which 866 patients (mean age, 70.1 years; male, 60.9%) with ischemic stroke (n = 781) or transient ischemic attack (n = 85) within 1 week of onset were consecutively enrolled and followed up for 1 year. The TyG index was calculated as ln (fasting triglycerides [mg/dL] × fasting glucose [mg/dL]/2). Patients were divided into 3 groups according to the tertile of TyG index levels: tertile 1, < 8.48; tertile 2, 8.48–9.01; and tertile 3, > 9.01. The primary outcome was a composite of major adverse cardiovascular events (MACE), including nonfatal stroke, nonfatal acute coronary syndrome, and vascular death.

**Results:**

The median TyG index was 8.74 (interquartile range, 8.34–9.16). Higher levels of TyG index were significantly associated with increased prevalence of ipsilateral extracranial carotid (*P* = 0.032) and intracranial (*P* = 0.003) atherosclerotic stenosis. There were significant differences in the MACE risk between the three groups (annual rate, 8.6%, 11.6%, and 17.3% in the tertile 1, tertile 2, tertile 3 groups, respectively; log-rank *P* = 0.005). After multivariable adjustments, the TyG index remains to be a significant predictor of MACE, with an adjusted hazard ratio for tertile 3 versus tertile 1 groups (95% confidence interval) of 2.01 (1.16–3.47). Similar results were also found for the risk of recurrent stroke.

**Conclusions:**

TyG index is associated with cervicocerebral atherosclerosis and the MACE risk after a stroke, suggesting the potential value of TyG index to optimize the risk stratification of stroke patients.

*Trial registration* URL: https://upload.umin.ac.jp. Unique identifier: UMIN000031913.

**Supplementary Information:**

The online version contains supplementary material available at 10.1186/s12933-022-01695-2.

## Background

Insulin resistance (IR), a hallmark of metabolic syndrome, is an important risk factor for stroke [1]. The current gold standard for measuring IR is the euglycemic hyperinsulinemic clamp test. However, given its complexity, invasiveness, and high cost, this test is not practical in clinical settings. The homeostasis model assessment of IR (HOMA-IR) has also been widely employed for research purposes to detect IR; however, it is substantially limited in clinical practice by the need to measure insulin levels. In addition, HOMA-IR cannot be applied to those who are being treated with insulin since exogenous insulin interferes with the value of the index.

Recently, a novel parameter named triglyceride-glucose (TyG) index, derived from fasting triglyceride (TG) and glucose levels, has been proposed as a simple and reliable surrogate marker of IR [2]. Prior studies have indicated that the diagnostic accuracy of the TyG index in detecting IR is comparable with that of the hyperinsulinemic euglycemic clamp test [3] or HOMA-IR [2, 4]. Furthermore, the TyG index is associated with the presence of coronary artery atherosclerosis, [5–7] and has been identified as an independent predictor of cardiovascular events in the general population [8–10] and in patients with acute coronary syndrome [11–13]. However, to date, few studies have addressed the correlation between the TyG index and ischemic stroke [14, 15]. Therefore, the primary aim of the present study was to investigate whether the TyG index can predict recurrent vascular events after stroke.

## Methods

### Study design and patients

The Tokyo Women’s Medical University (TWMU) Stroke Registry is an ongoing, prospective, single-center, observational study that enrolled patients with acute ischemic stroke or transient ischemic attack (TIA) within one week of onset, hospitalized at our center [16, 17]. The study conforms to the ethical guidelines of the 1975 Declaration of Helsinki, Ethical Guidelines for Epidemiological Research by the Japanese government, and Strengthening the Reporting of Observational Studies in Epidemiology (STROBE) guidelines. The study protocol was approved by the ethics committee of the Tokyo Women’s Medical University Hospital (approval no. 2955-R2). Written informed consent was obtained from all patients. The TWMU Stroke Registry is registered at UMIN000031913 (https://upload.umin.ac.jp). The data supporting the findings of this study are available from the corresponding author upon reasonable request.

**Fig. 1:**
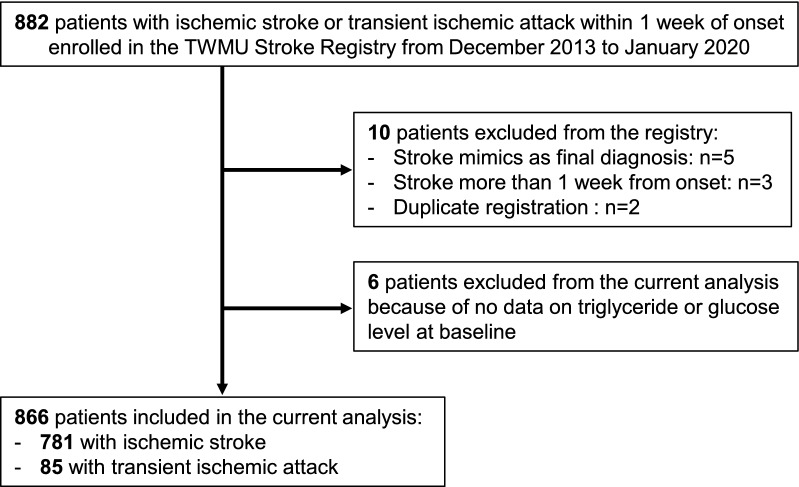
Study flow chart

Of 882 patients consecutively assessed for eligibility between December 2013 and January 2020, ten were excluded as their final diagnosis was a stroke mimic, more than one week had elapsed after stroke onset, or duplicate registrations were found. In addition, we excluded six patients who had no lipid or glucose profile data at baseline, leaving 866 patients (stroke, n = 781; TIA, n = 85) in the sample (Fig. 1).

All strokes and TIAs were diagnosed by board-certified stroke neurologists based on neurological and radiological findings. Patient data were collected for demographics, clinical symptoms during the qualifying event, medical history, investigations (including standard blood chemistry, brain and cerebral artery imaging, and cardiac work-up), management (medical treatment, revascularization procedure, and surgery), and the occurrence of clinical events after the qualifying event, using a structured case report form. Upon admission, neurological symptoms were assessed using the National Institutes of Health Stroke Scale (NIHSS) score.

### TyG index

The TyG index was calculated as ln (fasting TG [mg/dL] × fasting glucose [mg/dL]/2) [2].

The TG and glucose levels were assessed after overnight fasting within 48 h of admission. Because the normal range of TyG index has not been well defined, we divided the patients into three groups according to tertiles of the TyG index: tertile 1, < 8.48; tertile 2, 8.48–9.01; and tertile 3, > 9.01.

### Evaluation of vascular diseases

Intracranial arteries were examined using time-of-flight magnetic resonance angiography (n = 823) and/or computed tomography angiography (n = 183). The narrowest diameter of each stenosed vessel was measured and divided by the diameter of the normal vessel proximal to the lesion, or distal to the lesion if the proximal artery was diseased. Significant intracranial artery stenosis (ICAS) was defined as ≥ 50% stenosis or occlusion. ICAS was considered symptomatic if the stenosis was ipsilateral to the index stroke/TIA.

Extracranial carotid atherosclerosis was examined using ultrasonography (n = 813), and/or computed tomography angiography (n = 105), and/or time-of-flight magnetic resonance angiography (n = 78). We defined significant extracranial artery stenosis (ECAS) as the presence of atherosclerotic stenosis of ≥ 50% or occlusion according to the European Carotid Surgery Trial criteria [18]. ECAS was considered symptomatic if the stenosis was ipsilateral to the index stroke/TIA.

Aortic atherosclerosis was assessed using transesophageal echocardiography (n = 260). Mobile plaques were defined as mobile components seen swinging on their peduncles. An ulcerative plaque was diagnosed as a discrete indentation of the luminal surface of the plaque with a base width and maximum depth of at least 2 mm each. Complex aortic atheroma were defined as any plaque ≥ 4 mm in thickness or a plaque with ulceration or mobile components [19].

Cerebral small vessel disease was assessed using fluid-attenuated inversion recovery images acquired using a 1.5 T magnetic resonance imaging scanner (Philips Ingenia 1.5 T, Siemens Magnetom Avanto fit 1.5 T) (n = 821). Degrees of periventricular hyperintensity and deep white matter hyperintensity were rated according to a published definition (0–4 for each hemisphere) [20]. The higher score between the right and left sides was used in the analysis.

### Etiologic subtype

An etiologic subtype of the index event was assigned to each patient using the atherosclerosis, small vessel disease, cardiac pathology, other definite causes, and dissection (ASCOD) grading system [21]. The ASCOD system categorizes five predefined phenotypes. Each of the phenotypes is graded according to if the disease is: (1) likely causal; (2) potentially causal but uncertain; (3) present but is unlikely to be causal, and (0) absent. We defined stroke of atherothrombotic origin as ASCOD grade A1 or A2, which corresponds to the presence of symptomatic or potentially symptomatic ipsilateral ICAS or ECAS or complex aortic atheroma. Similarly, small vessel disease (lacunar stroke) and cardioembolic stroke were defined as ASCOD grades S1 or S2, and C1 or C2, respectively.

### Follow-up and outcomes

Follow-up visits were scheduled at three months, one year, and three years after enrollment. The present study reports the one-year outcomes. At each follow-up visit, treatments, occurrence of clinical events, and modified Rankin Scale scores were recorded. If the patient could not be contacted for follow-up, a relative or caregiver was interviewed via telephone. The primary outcome was a composite of major adverse cardiovascular events (MACE), including nonfatal stroke (either ischemic or hemorrhagic), nonfatal acute coronary syndrome, major peripheral artery disease, and vascular death. Vascular death was defined as fatal acute coronary syndrome, fatal stroke, and other cardiovascular deaths including pulmonary embolism and sudden cardiac death. The secondary outcomes included the presence of cervicocerebral artery stenosis and the occurrence of any stroke (either ischemic or hemorrhagic) and all-cause death.

### Statistical analysis

Continuous variables are expressed as mean (standard deviation) in cases of normal distribution, or median (interquartile range). Categorical variables are expressed as frequencies (percentages). Comparisons were made between multiple groups using the Jonckheere-Terpstra test for continuous variables, the Cochrane-Armitage test for categorical variables, and the log-rank test for censored variables. Event rates were estimated using the Kaplan–Meier method. Cox proportional hazard regression models were used to evaluate the associations between the TyG index and risk of recurrent vascular events by calculating hazard ratios and 95% confidence intervals (CIs). Age and all variables with *P* < 0.10 in univariable analysis (i.e., sex, body mass index, hypertension, dyslipidemia, diabetes mellitus, atrial fibrillation, chronic heart failure, current smoking, excessive alcohol, and etiologic subtype of index event) were included in the multivariable adjustments. Data for patients with no information at one year were censored at the time of the last available follow-up. For a given outcome, patients who died from causes other than the outcome were censored at the time of death. The Gray’s test was applied in the analysis of the cumulative incidence by treating death as a competing risk. Sensitivity analyses were conducted to assess the effect of TyG on vascular risk according to the etiological subtype of the index event. The area under the receiver operating characteristic curve (AUC) was used to describe the predictive value of the TyG index for MACE. The optimal cut-off was determined using the Youden index. For all analyses, statistical significance was set at *P* < 0.05. Statistical analysis was performed with JMP Pro version 16 (SAS institute, Cary, NC) and EZR version 1.61 (Saitama Medical Center, Jichi Medical University, Saitama, Japan).

## Results

A total of 866 patients (mean age, 70.1 years; male, 60.9%) were included in the analysis. The median TyG index was 8.74 (interquartile range, 8.34–9.16). Patients were classified into three groups according to tertiles of the TyG index (tertile 1, TyG index < 8.48, n = 288; tertile 2, 8.48 ≤ TyG index ≤ 9.01, n = 289; and tertile 3, TyG index > 9.01, n = 289). The mean (standard deviation) TyG index levels of the three groups were 8.11 (0.33), 8.74 (0.15), and 9.41 (0.49), respectively. Table 1 presents the baseline patient characteristics. Compared with patients in the lowest tertile group, those in the higher tertile groups were more likely to be male and obese, to have hypertension, dyslipidemia, diabetes, and drinking habit, and less likely to have atrial fibrillation. The examination and investigation results are presented in (Additional file 1: Table S1). Higher TyG index levels were associated with higher systolic blood pressure, low-density lipoprotein cholesterol, TG, glucose, and HbA1c levels, and lower high-density lipoprotein cholesterol levels. Regarding the etiologic subtype of the index event, patients in the highest tertile group were more likely to have had atherothrombosis, whereas those in the lowest tertile group were more likely to have had cardioembolism.

**Table 1:** Baseline characteristics

	Total (n = 866)	TyG index			*P* value for trend
		Tertile 1 (n = 288)	Tertile 2 (n = 299)	Tertile 3 (n = 299)	
Age, years, mean (SD)	70.1 (13.7)	70.9 (14.9)	70.1 (13.6)	69.3 (12.4)	0.21
Male, n (%)	527 (60.9)	159 (55.2)	174 (60.2)	194 (67.1)	0.003
BMI, kg/m^2^, mean (SD)	23.2 (4.1)	21.9 (3.7)	23.0 (3.9)	24.5 (4.2)	< 0.001
Medical history, n (%)					
Hypertension	622 (71.9)	195 (67.7)	202 (70.1)	225 (77.9)	0.007
Dyslipidemia	392 (45.3)	92 (31.9)	137 (47.4)	163 (56.4)	< 0.001
Diabetes mellitus	320 (37.0)	52 (18.1)	92 (31.9)	176 (60.9)	< 0.001
Chronic kidney disease	233 (26.9)	75 (26.0)	74 (25.6)	84 (29.1)	0.41
Atrial fibrillation	195 (22.5)	81 (28.1)	67 (23.2)	47 (16.3)	< 0.001
Chronic heart failure	112 (12.9)	47 (16.3)	31.3 (12.1)	30 (10.4)	0.034
Stroke or TIA	170 (19.6)	55 (19.1)	54 (18.7)	61 (21.1)	0.54
Coronary artery disease	115 (13.3)	30 (10.4)	42 (14.5)	43 (14.9)	0.11
Peripheral artery disease	45 (5.2)	13 (4.5)	12 (4.2)	20 (6.9)	0.19
Current smoking	150 (17.3)	40 (13.9)	48 (16.6)	62 (21.5)	0.016
Excessive alcohol	56 (6.5)	14 (4.9)	11 (3.8)	31 (10.7)	0.004

Patients were divided into three groups according to the tertile of TyG index: tertile 1, < 8.48; tertile 2, 8.48–9.01; and tertile 3, > 9.01

*BMI* indicates body mass index; *SD* standard deviation; *TIA* transient ischemic attack; *TyG* triglyceride-glucose

### TyG index and vascular diseases

**Fig. 2:**
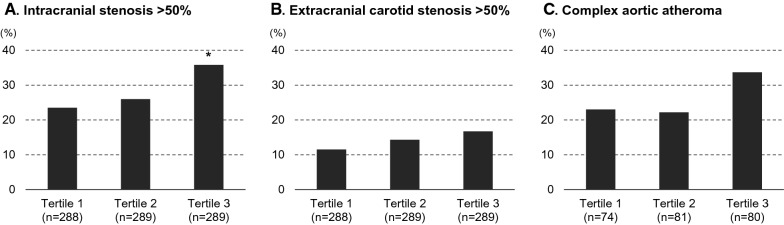
**Prevalence of atherosclerotic diseases** * *P* < 0.05. Prevalence of intracranial stenosis > 50% (**A**), extracranial carotid stenosis > 50% (**B**), and complex aortic atheroma (**C**). Patients were divided into three groups according to the tertile of TyG index: tertile 1, < 8.48; tertile 2, 8.48–9.01; and tertile 3, > 9.01.

**Fig. 3:**
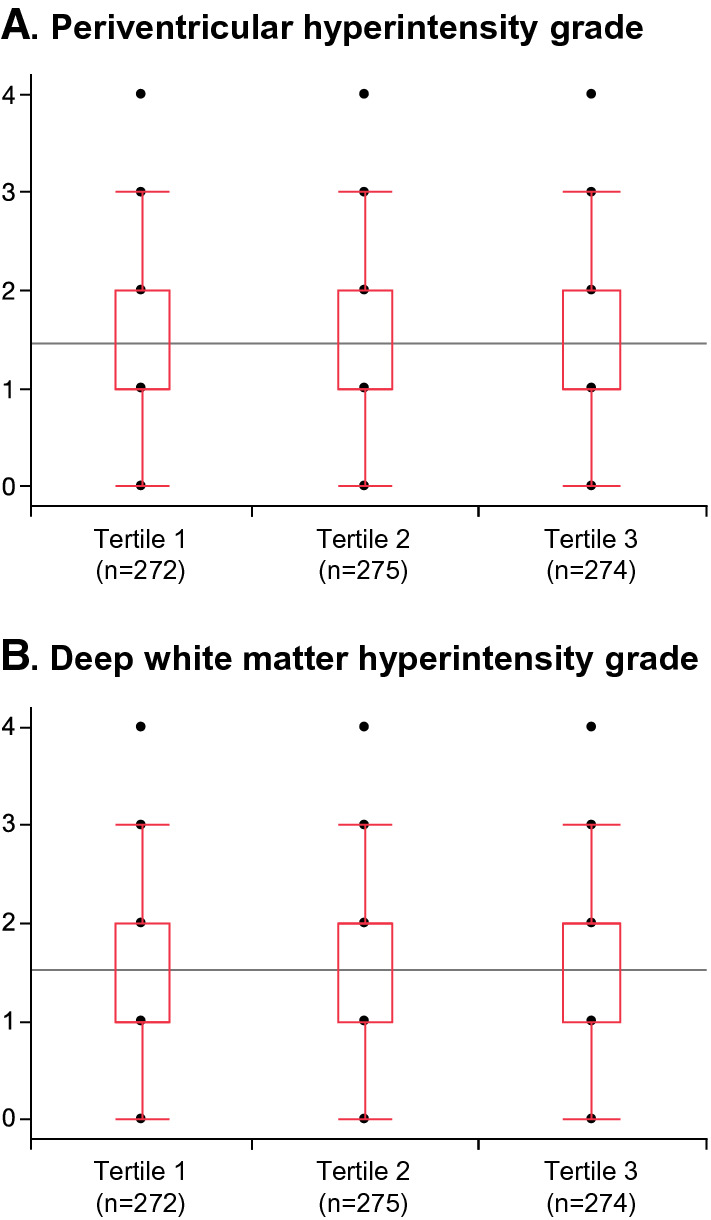
**Degree of small vessel disease on magnetic resonance imaging** Patients were divided into three groups according to the tertile of TyG index: tertile 1, < 8.48; tertile 2, 8.48–9.01; and tertile 3, > 9.01. **A** Medians (interquartile ranges) of periventricular hyperintensity grade were 1 (1–2), 1 (1–2), and 1 (1–2) in the terteile 1, tertile 2, tertile 3 groups, respectively, with no significant differences between the groups (*P* for trend = 0.49). **B** Medians (interquartile ranges) of deep white matter hyperintensity grade were 1 (1–2), 2 (1–2), and 2 (1–2) in the terteile 1, tertile 2, tertile 3 groups, respectively, with no significant differences between the groups (*P* for trend = 0.19).

Figure 2 and Additional file 2: Figure S1 show the prevalence of atherosclerotic diseases in the intracranial and extracranial arteries and aorta. Patients in the highest tertile group were more likely to have ICAS (adjusted odds ratio, 1.66; 95% CI 1.07–2.57) and symptomatic ECAS (adjusted odds ratio, 1.94; 95% CI 1.00–3.74) than those in the lowest tertile group. As shown in Fig. 3, there were no significant associations between the TyG index and the degree of small vessel disease on magnetic resonance imaging.

### One-year outcomes

The treatments at discharge are presented in Additional file 3: Table S2. At discharge, lipid-lowering and antidiabetic agents were used in 62.3% and 28.6% of all patients, respectively, and patients with a higher TyG index were more likely to receive these medications. Thirty-two (3.7%) patients were lost to the one-year follow-up.

**Fig. 4:**
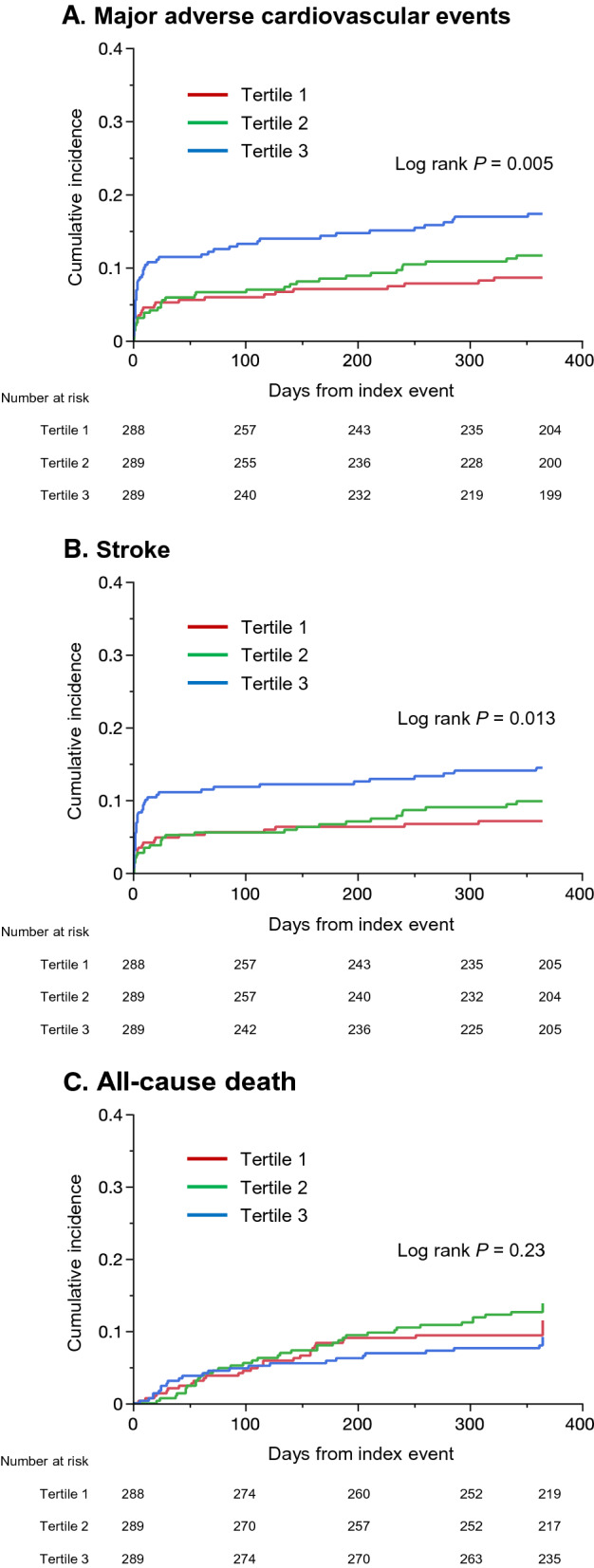
**Kaplan–Meier event curves** Cumulative event rates of major adverse cardiovascular events (**A**) and stroke (**B**). Patients were divided into three groups according to the tertile of TyG index: tertile 1, < 8.48; tertile 2, 8.48–9.01; and tertile 3, > 9.01.

**Fig. 5:**
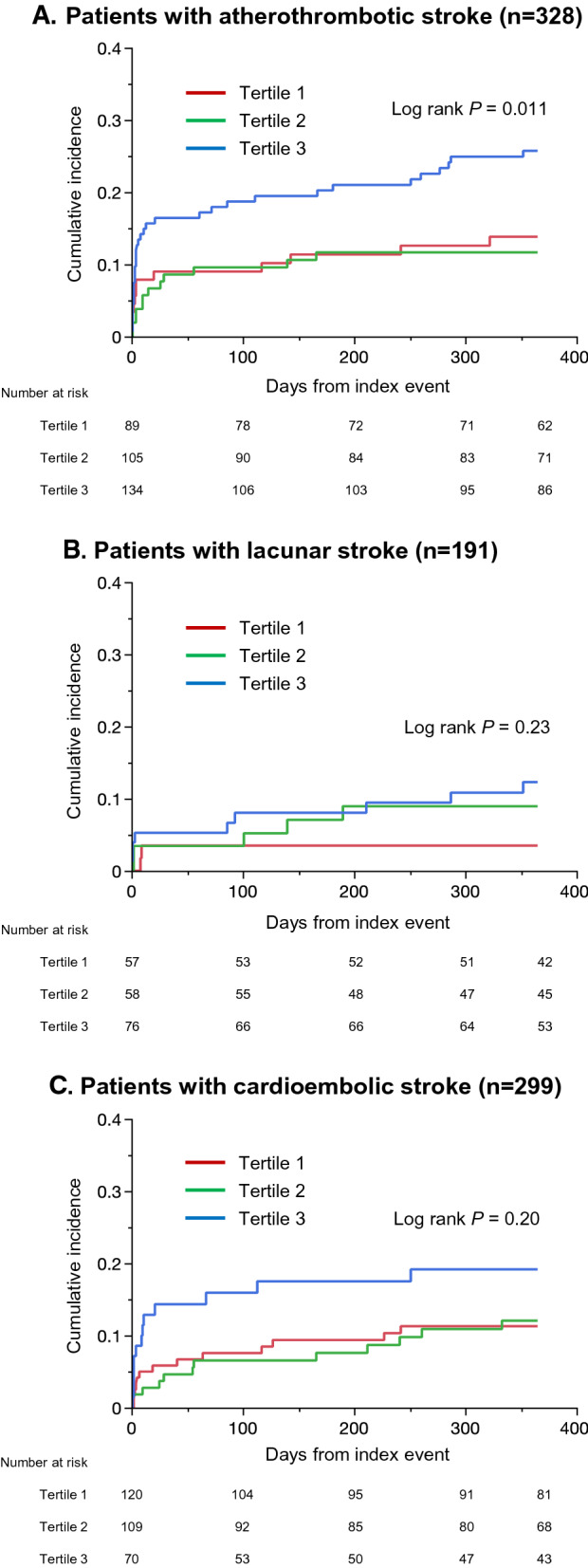
**Kaplan–Meier event curves according to the etiologic subtype of index event** Cumulative event rates of major adverse cardiovascular events in patients with atherothrombotic (**A**), lacunar (**B**), and cardioembolic (**C**) strokes. Patients were divided into three groups according to the tertile of TyG index: tertile 1, < 8.48; tertile 2, 8.48–9.01; and tertile 3, > 9.01.

Among the 866 patients, 105 had at least one vascular event within one year, resulting in an event rate of 12.5% (95% CI 10.4–14.9%). As shown in Fig. 4 and Table 2, patients in the highest tertile group had a significantly higher risk of MACE (adjusted hazard ratio, 1.93; 95% CI, 1.12–3.33) and stroke (adjusted hazard ratio, 1.92; 95% CI, 1.05–3.49) than those in the lowest tertile group. In the Gray’s test, the differences were still significant for both MACE (*P* = 0.001) and stroke (*P* = 0.004). The optimal TyG index cut-off for predicting MACE was 8.98 (sensitivity, 67.5%; specificity, 49.6%; AUC, 0.579). There was no difference in the risk of all-cause death among the three groups. When patients were classified according to the etiologic subtype of the index event (Fig. 5), the TyG index was associated with a higher MACE risk among patients with atherothrombotic stroke (log-rank *P* = 0.011), whereas no differences were observed among those with small-vessel disease (log-rank *P* = 0.23) or cardioembolic stroke (log-rank *P* = 0.20).

**Table 2:** Event risk at one year

Outcome	TyG index	Event, n (%)	Unadjusted HR (95% CI)	*P* value	Adjusted HR (95% CI)^a^	*P* value^a^
MACE	Tertile 1	24 (8.6)	1.00 (ref)	–	1.00 (ref)	–
	Tertile 2	32 (11.6)	1.34 (0.79–2.27)	0.28	1.35 (0.78–2.32)	0.28
	Tertile 3	49 (17.3)	2.13 (1.30–3.47)	0.003	1.93 (1.12–3.33)	0.018
Stroke	Tertile 1	20 (7.1)	1.00 (ref)	–	1.00 (ref)	–
	Tertile 2	27 (9.8)	1.35 (0.75–2.40)	0.31	1.35 (0.74–2.45)	0.33
	Tertile 3	41(14.5)	2.12 (1.24–3.62)	0.006	1.92 (1.05–3.49)	0.033
All-cause death	Tertile 1	32 (11.5)	1.00 (ref)	–	1.00 (ref)	–
	Tertile 2	39 (13.8)	1.22 (0.77–1.95)	0.40	1.32 (0.81–2.16)	0.27
	Tertile 3	26 (9.2)	0.80 (0.48–1.34)	0.40	0.71 (0.40–2.39)	0.22

Patients were divided into three groups according to the tertile of TyG index: tertile 1, < 8.48; tertile 2, 8.48–9.01; and tertile 3, > 9.01

MACE indicates major adverse cardiovascular events; TyG, triglyceride-glucose

^a^Adjusted for age, sex, body mass index, hypertension, dyslipidemia, diabetes mellitus, atrial fibrillation, chronic heart failure, current smoking, excessive alcohol, and etiologic subtype of index event

## Discussion

In this prospective observational registry study involving Japanese patients with recent stroke or TIA, the TyG index was significantly associated with prevalence of intra- and extracranial artery atherosclerosis and was an independent predictor of future MACE. The prognostic value of the TyG index differed between the etiologic subtypes of stroke; a significant correlation between the TyG index and MACE risk was observed in atherothrombotic stroke but not in lacunar or cardioembolic stroke. Our results suggest the usefulness of TyG index as a simple risk predictor, allowing for more targeted secondary prevention.

It is plausible that IR contributes to the development of vascular events through multiple mechanisms. The biological consequences of IR include hypertension, dyslipidemia, hyperglycemia, hyperinsulinemia, systemic inflammation, and vascular endothelial dysfunction, all of which can accelerate atherosclerosis [1]. IR also promotes platelet adhesion, activation, and aggregation, [22] leading to the occurrence of thrombotic events. In addition, IR can cause impairments of cerebral vascular reactivity and hemodynamic disturbances that may facilitate the onset of stroke [23]. These mechanisms are able to explain our results, although the causal relationship between TyG index and vascular events needs to be confirmed in further studies.

The TyG index can be easily and inexpensively obtained in clinical practice using routine blood tests. A previous study showed that the TyG index is a highly sensitive (96.5%) and specific (85.0%) method to detect IR, compared to the euglycemic hyperinsulinemic technique [3]. In addition, the TyG index has been shown to be superior to the HOMA-IR in assessing IR in individuals with or without diabetes [2, 4]. After the TyG index was first proposed in 2008, [2] a number of clinical studies found significant associations of the TyG index with arterial stiffness [24, 25] and the presence of atherosclerotic diseases [5–7]. Furthermore, epidemiological studies have demonstrated that the TyG index is predictive of future stroke in the general population [8–10]. According to a meta-analysis including more than 5 million participants, the TyG index was independently associated with 1.4-fold and 1.3-fold increased risk of coronary artery disease and stroke, respectively [8]. Another cohort study found a linear relationship between the TyG index and the risk of ischemic stroke, but not hemorrhagic stroke [9]. In prior studies targeting acute stroke patients, the TyG index was associated with early recurrent ischemic lesions on brain imaging, [14] early neurological worsening, [15] and recurrent stroke, [15] which is in agreement with our results. The predictive performance of TyG index observed in our study (sensitivity, 67.5%; specificity, 49.6%; AUC, 0.579) seems to be similar to that in previous studies reporting the sensitivity of 46.0–59.2%, specificity of 63.2–68.5%, and AUC of 0.560–0.633 to predict MACE in the general population [6, 26] or coronary artery disease patients [27]. We assumed that the prognostic impact of the TyG index may differ between etiologic subtypes of stroke, given the adverse effects of IR on the atherosclerotic process. As expected, the TyG index was strongly associated with the presence of ICAS and ECAS and with the risk of recurrent vascular events after atherothrombotic stroke or TIA.

### Limitations

This study has several limitations. First, because the study participants were from a single-center cohort of Japanese patients, our results may not be generalizable to other populations. The annual MACE rate of 12.5% was higher than that reported in previous clinical trials [28, 29]. This may be due to our consecutive enrollment of patients, regardless of their age, general condition, or comorbidities, whereas clinical trials usually select patients whose general condition is fair. In addition, patients were included within one week of onset, ensuring that early recurrent events were captured. Second, the TyG index measured during the acute phase of stroke might not precisely reflect the patient’s insulin sensitivity, given that acute stroke could lead to stress hyperglycemia. Moreover, we did not assess temporal changes in the index during the follow-up period. Some prior studies have measured the TyG index several times between specific intervals and demonstrated that an index of cumulative exposure to TyG is superior to a single measurement in risk prediction [30, 31]. Hence, the use of the TyG index only at baseline may be less robust. Third, we could not calculate the HOMA-IR because our database lacked data on fasting insulin levels. It would be interesting to compare the predictive ability of the TyG index with that of the HOMA-IR. Forth, among the 105 MACEs observed at one year, 37 (35.2%) occurred during hospitalization. Medications at discharge shown in Additional file 3: Table S2 do not necessarily reflect the treatments when the patients had recurrent events. Finally, although the TyG index was an independent predictor, its sensitivity (67.5%) and specificity (49.6%) are still poor, suggesting that it is difficult to predict MACE based on the TyG index alone. The predictive performance of the TyG index needs to be validated in larger cohort studies.

## Conclusions

The present study indicated independent associations of the TyG index, a surrogate for IR, with the risk of MACE and recurrent stroke in patients with recent stroke or TIA. The TyG index may be useful as a convenient marker for optimizing risk stratification for stroke or TIA.

## Supplementary Information

**Additional file 1: Table S1.** Examination and investigation findings.

**Additional file 2: Figure S1.** Prevalence of atherosclerotic diseases.

**Additional file 3: Table S2.** Medication use at discharge.

## Data Availability

The data supporting the findings of this study are available from the corresponding author upon reasonable request.

## Abbreviations

ASCOD: Atherosclerosis, small vessel disease, cardiac pathology, other definite causes, and dissection
AUC: Area under the receiver operating characteristic curve
CI: Confidence interval
ECAS: Extracranial artery stenosis
HOMA-IR: Homeostasis model assessment of insulin resistance
ICAS: Intracranial artery stenosis
MACE: Major adverse cardiovascular events
NIHSS: National Institute of Health Stroke Scale
STROBE: Strengthening the Reporting of Observational Studies in Epidemiology
TG: Triglyceride
TIA: Transient ischemic attack
TWMU: Tokyo Women’s Medical University
TyG: Triglyceride-glucose
