# Supplementary material for: Triglyceride-glucose index as a prognostic marker after ischemic stroke or transient ischemic attack: a prospective observational study
Source: Cardiovasc Diabetol. 2022 Nov 30;21:264. doi: 10.1186/s12933-022-01695-2 (PMC9714168; doi:10.1186/s12933-022-01695-2)
Supplement: Supplementary file 1 — Additional file 1: Table S1. Examination and investigation findings. [file 12933_2022_1695_MOESM1_ESM.pdf]

Supplementary Table 1. Examination and investigation findings

	Total (n=866)	TyG index			*P* value for trend
		Tertile 1 (n=288)	Tertile 2 (n=299)	Tertile 3 (n=299)	
Blood pressure					
Systolic, mm Hg, mean (SD)	152 (27)	149 (27)	151 (28)	155 (27)	0.040
Diastolic, mm Hg, mean (SD)	86 (17)	85 (18)	86 (16)	87 (18)	0.17
Laboratory data					
LDL-C, mg/dL, mean (SD)	116 (38)	108 (32)	114 (40)	125 (39)	<0.001
HDL-C, mg/dL, mean (SD)	56 (18)	64 (18)	56 (17)	48 (14)	<0.001
TG, mg/dL, median (IQR)	109 (77–149)	70 (56–83)	113 (92–129)	169 (137–227)	<0.001
Glucose, mg/dL, median (IQR)	113 (99–139)	101 (91–112)	112 (99–130)	142 (116–192)	<0.001
HbA1c, %, median (IQR)	6.0 (5.6–6.9)	5.8 (5.5–6.1)	6 (5.6–6.6)	6.9 (6.0–8.1)	<0.001
Initial NIHSS, median (IQR)	3 (1–5)	2 (1–4)	3 (1–6)	3 (1–5)	0.14
ASCOD grade, n(%)					
Atherothrombosis					
A1 or A2	328 (37.9)	89 (30.9)	105 (36.3)	134 (46.4)	<0.001
A3	200 (23.1)	67 (23.3)	38 (23.5)	65 (22.5)	0.83
A0	334 (38.6)	129 (44.8)	115 (39.8)	90 (31.1)	<0.001
Small vessel disease					
S1 or S2	191 (22.1)	57 (19.8)	58 (20.1)	76 (26.3)	0.030
S3	251 (29.0)	81 (28.1)	82 (28.4)	88 (30.5)	0.54
S0	424 (49.0)	150 (52.1)	149 (51.6)	125 (43.3)	0.034
Cardiac pathology					
C1 or C2	299 (34.5)	120 (41.7)	109 (37.7)	70 (24.2)	<0.001
C3	126 (14.6)	34 (11.8)	31 (10.7)	61 (21.1)	<0.001
C0	441 (50.9)	134 (46.5)	149 (51.6)	158 (54.7)	0.050

Patients were divided into three groups according to the tertile of TyG index: tertile 1, <8.48; tertile 2, 8.48–9.01; and tertile 3, >9.01.
ASCOD indicates atherosclerosis, small vessel disease, cardiac pathology, other definite cause; HDL-C, high-density lipoprotein cholesterol; IQR, interquartile range; LDL-C, low-density lipoprotein cholesterol; NIHSS, National Institute of Health Stroke Scale; SD, standard deviation; TG, triglycerides; TyG, triglyceride-glucose.
