# Supplementary material for: Triglyceride-glucose index as a prognostic marker after ischemic stroke or transient ischemic attack: a prospective observational study
Source: Cardiovasc Diabetol. 2022 Nov 30;21:264. doi: 10.1186/s12933-022-01695-2 (PMC9714168; doi:10.1186/s12933-022-01695-2)
Supplement: Supplementary file 2 — Additional file 2: Figure S1. Prevalence of atherosclerotic diseases. [file 12933_2022_1695_MOESM2_ESM.pdf]

Supplementary Figure 1. Prevalence of atherosclerotic diseases

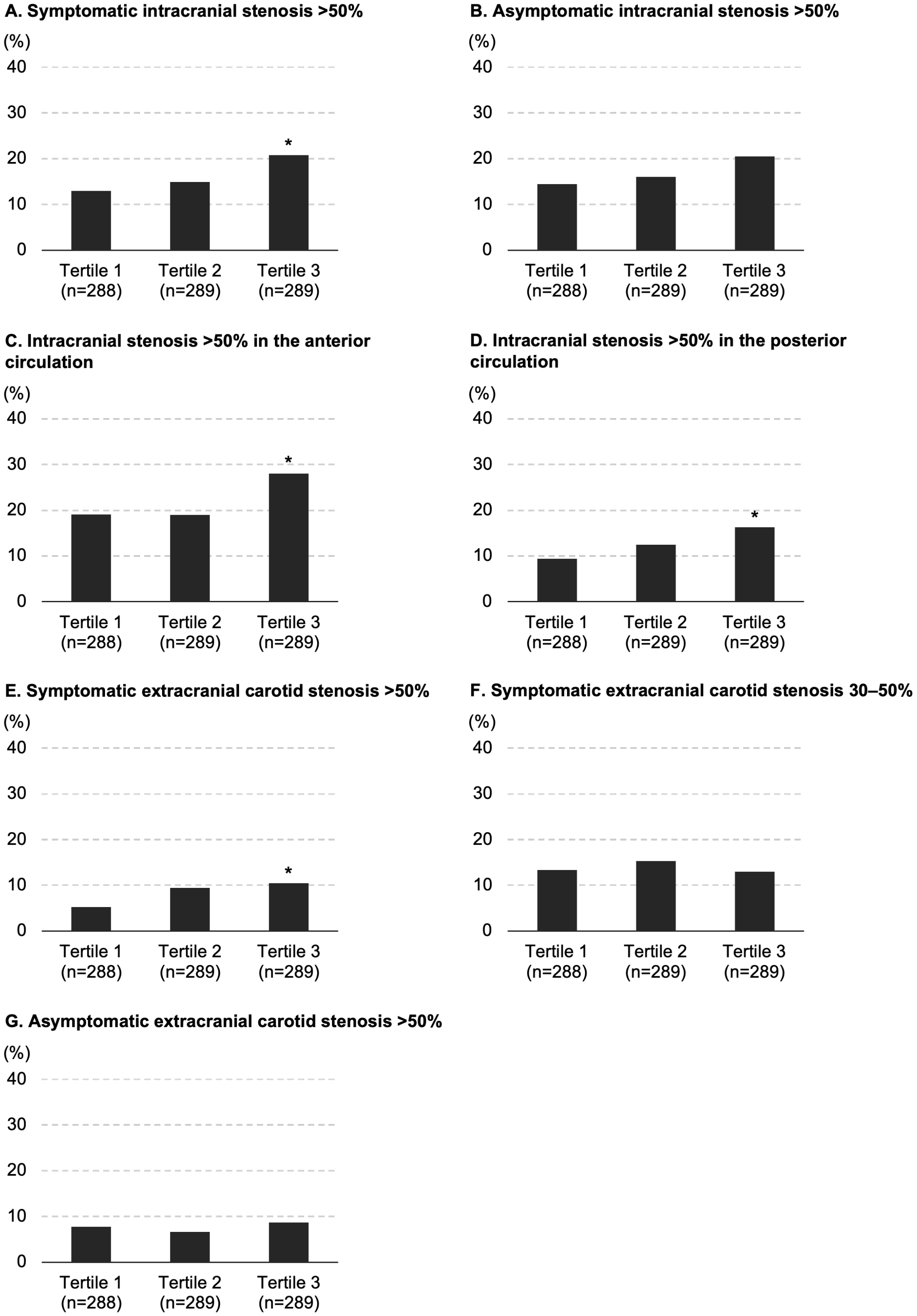

* $P<0.05$

Patients were divided into three groups according to the tertile of TyG index: tertile 1, <8.48; tertile 2, 8.48–9.01; and tertile 3, >9.01.
