# Supplementary material for: Triglyceride-glucose index as a prognostic marker after ischemic stroke or transient ischemic attack: a prospective observational study
Source: Cardiovasc Diabetol. 2022 Nov 30;21:264. doi: 10.1186/s12933-022-01695-2 (PMC9714168; doi:10.1186/s12933-022-01695-2)
Supplement: Supplementary file 3 — Additional file 3: Table S2. Medication use at discharge. [file 12933_2022_1695_MOESM3_ESM.pdf]

Supplementary Table 2. Medication use at discharge

	Total (n=866)	TyG index			*P* value for trend
		Tertile 1 (n=288)	Tertile 2 (n=299)	Tertile 3 (n=299)	
Any antiplatelet agents	592 (70.4)	173 (62.7)	197 (68.9)	222 (79.6)	<0.001
Dual antiplatelet therapy	231 (28.7)	59 (22.3)	74 (27.3)	98 (36.6)	0.001
Any anticoagulant agents	291 (34.6)	112 (40.6)	109 (38.3)	70 (25.1)	<0.001
Direct oral anticoagulant	160 (18.5)	56 (19.4)	60 (20.8)	44 (15.2)	0.19
Warfarin	119 (13.7)	56 (19.4)	42 (14.5)	21 (7.3)	<0.001
Any antihypertensive agents	482 (56.3)	156 (55.1)	156 (54.7)	170 (59.2)	0.48
Calcium channel blocker	276 (31.9)	87 (30.2)	91 (31.5)	98 (33.9)	0.63
Angiotensin receptor II blocker	250 (29.2)	77 (27.0)	81 (28.2)	92 (32.3)	0.35
Any lipid-lowering agents	529 (62.3)	146 (52.5)	166 (58.0)	217 (76.1)	<0.001
Statin	485 (56.0)	137 (47.6)	162 (56.1)	186 (64.4)	<0.001
Any antidiabetic agents	241 (28.6)	33 (12.0)	60 (21.1)	148 (52.1)	<0.001
Insulin	109 (12.6)	16 (5.6)	29 (10.0)	64 (22.2)	<0.001
Dipeptidyl peptidase-4 inhibitor	144 (16.6)	17 (5.9)	34 (11.8)	93 (32.2)	<0.001
Biguanide	56 (6.5)	4 (1.4)	14 (4.8)	38 (13.2)	<0.001
Sulfonylurea	50 (5.8)	8 (2.8)	7 (2.4)	35 (12.1)	<0.001
Alpha-glucosidase inhibitor	37 (4.3)	3 (1.0)	12 (4.2)	22 (7.6)	<0.001
Thiazolidinedione	6 (0.7)	1 (0.4)	0	5 (1.7)	0.020

Shown are n (%).

Patients were divided into three groups according to the tertile of TyG index: tertile 1, <8.48; tertile 2, 8.48–9.01; and tertile 3, >9.01.
